# *Staphylococcus aureus* Extracellular Vesicles: A Story of Toxicity and the Stress of 2020

**DOI:** 10.3390/toxins13020075

**Published:** 2021-01-20

**Authors:** Xiaogang Wang, Paul F. Koffi, Olivia F. English, Jean C. Lee

**Affiliations:** Brigham and Women’s Hospital and Harvard Medical School, Boston, MA 02115, USA; xwang39@bwh.harvard.edu (X.W.); pkoffi@bwh.harvard.edu (P.F.K.); oenglish@bwh.harvard.edu (O.F.E.)

**Keywords:** *Staphylococcus aureus*, extracellular vesicles, toxins, stress

## Abstract

*Staphylococcus aureus* generates and releases extracellular vesicles (EVs) that package cytosolic, cell-wall associated, and membrane proteins, as well as glycopolymers and exoproteins, including alpha hemolysin, leukocidins, phenol-soluble modulins, superantigens, and enzymes. *S. aureus* EVs, but not EVs from pore-forming toxin-deficient strains, were cytolytic for a variety of mammalian cell types, but EV internalization was not essential for cytotoxicity. Because *S. aureus* is subject to various environmental stresses during its encounters with the host during infection, we assessed how these exposures affected EV production in vitro. Staphylococci grown at 37 °C or 40 °C did not differ in EV production, but cultures incubated at 30 °C yielded more EVs when grown to the same optical density. *S. aureus* cultivated in the presence of oxidative stress, in iron-limited media, or with subinhibitory concentrations of ethanol, showed greater EV production as determined by protein yield and quantitative immunoblots. In contrast, hyperosmotic stress or subinhibitory concentrations of erythromycin reduced *S. aureus* EV yield. EVs represent a novel *S. aureus* secretory system that is affected by a variety of stress responses and allows the delivery of biologically active pore-forming toxins and other virulence determinants to host cells.

## 1. Introduction

*Staphylococcus aureus* is a pathogenic bacterium that causes a wide spectrum of human diseases, ranging from mild skin lesions and surgical wound infections, to invasive and life-threatening infections, such as pneumonia, osteomyelitis, endocarditis, and bacteremia [1]. Many *S. aureus* isolates are resistant to commonly used antibiotics, and efforts to develop a vaccine for the prevention of staphylococcal infections have eluded success [2]. The pathogenesis of *S. aureus* infections is attributed to a wide array of virulence determinants that are associated with the cell surface, such as protein adhesins [3] and glycopolymers [4], or secreted to the environment, such as pore-forming toxins (PFTs) [5], superantigens [6], and proteases [7].

*S. aureus* toxins are mainly expressed and secreted during the post-exponential phase of bacterial growth. These exoproteins enhance bacterial virulence by directly lysing host cells, orchestrating intracellular signaling events, or activating T cells, thereby playing important roles in the pathogenesis of staphylococcal disease [8,9]. *S. aureus* toxins can be divided into three categories according to their effects on host cells: (1) PFTs lyse host cells by forming pores in the plasma membrane in a receptor-dependent manner [5]. (2) Phenol-soluble modulins (PSMs) and delta hemolysin, a group of small amphipathic peptides with alpha-helical structures, lyse host cells by nonspecific destruction of cytoplasmic membranes due to their surfactant-like characteristics [10]. (3) Pyrogenic toxins, including enterotoxins and toxic shock syndrome toxin-1, are superantigens that cross link the Vβ region of the human T cell receptor with MHC Class II on antigen-presenting cells, resulting in the activation of up to 30% of T cells and leading to increased T-cell proliferation and a cytokine storm. Enterotoxin production by *S. aureus* strains in nonimmune hosts may lead to toxic shock syndrome, a life-threatening disease that is characterized by rash, hypotension, fever, and multiorgan dysfunction. Human consumption of food containing preformed enterotoxins may result in acute food poisoning [11].

In addition to toxins, *S. aureus* also produces a variety of extracellular proteases, which can promote bacterial invasion and dissemination or dampen host innate immunity by directly degrading host proteins [12,13,14]. Staphylococcal proteases also modulate the stability of bacterial-derived virulence determinants [7,15]. Except for PSMs that are exported by an ATP-binding cassette transporter [16], most *S. aureus* exoproteins are secreted through the general secretory (Sec) pathway [17]. Toxins that are released from the bacterial cell as soluble molecules into the surrounding milieu are subject to destruction by host or bacterial proteases or neutralization by toxin-specific antibodies.

**Figure 1:**
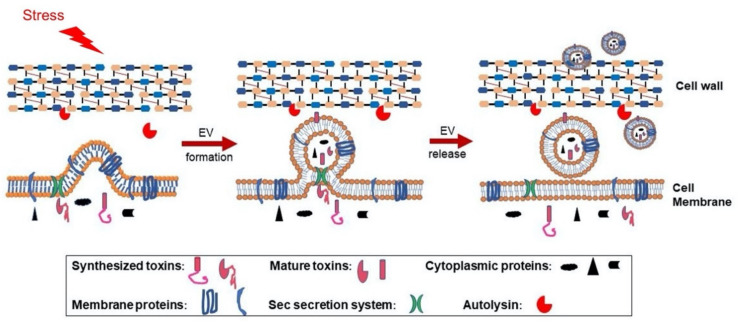
Proposed model for *S. aureus* extracellular vesicle (EV) biogenesis. EVs are formed from the bacterial cytoplasmic membrane due to turgor pressure, and this process is modulated by environmental stresses or by *S. aureus* PSMα peptides, which have surfactant-like activity, enhancing membrane curvature and EV formation. During biogenesis, selective cytoplasmic proteins and membrane proteins are packaged within EVs. Although *S. aureus* exoproteins are normally processed by the Sec machinery and released into the culture supernatant, the bacterial secretome includes EV toxin cargo. If exoproteins are transported to the EV lumen or associate with the membrane when Sec-mediated secretion occurs at the junction between the EV and the mother cell, the toxins are likely to lose their signal peptides, fold properly, and demonstrate biological activity. EVs released from the cytoplasmic membrane must traverse the highly cross-linked bacterial cell wall before release, and this process is promoted by *S. aureus* autolysins, such as Sle1 [24], which reduce cell wall crosslinking by hydrolyzing specific linkages within the peptidoglycan. PSM: Phenol-soluble modulins.

Extracellular vesicles (EVs) are nano-sized, spherical, bi-layered membrane vesicles that are secreted by eukaryotes, archaea, and bacteria [18]. The generation of EVs from multiple *S. aureus* strains, including antibiotic-resistant isolates, has been characterized during the past decade [19,20,21,22,23,24]. *S. aureus* EVs package a diverse array of bacterial components, including cytosolic, surface, and membrane proteins, as well as surface adhesins, lipoproteins, and toxins [19,20,21,24]. The mechanisms underlying EV production in Gram-positive bacteria are poorly understood. Because of the single membrane and the thick peptidoglycan structure typical of Gram-positive microbes, the biogenesis of *S. aureus* EVs is a complex process. We demonstrated that alpha-type phenol-soluble modulins promote EV biogenesis by disrupting the bacterial cytoplasmic membrane, whereas peptidoglycan crosslinking and autolysin activity modulate EV production by altering the permeability of the cell wall [24] (Figure 1). EVs purified from multiple *S. aureus* isolates exhibit dose-dependent cellular toxicity [19,20,24,25,26] since EV cargo includes multiple PFTs [20,23,24,25,27,28,29]. Analysis of EV protein content by mass spectrometry revealed that EV-associated *S. aureus* toxins do not include their signal sequence, consistent with the observation that the toxins are packaged as biologically active molecules. Most *S. aureus* exoproteins are synthesized in the cytoplasm as preproteins with an N-terminal signal peptide. Recognition of the signal sequence by the Sec machinery leads to translocation of the preprotein across the cell membrane in an unfolded state. Following cleavage of the signal peptide by a type I signal peptidase [30], the protein folds into its native conformation within the space between the membrane and the cell wall, assisted by chaperone proteins [17]. Because *S. aureus* EVs are generated by a budding process from the cell membrane under turgor pressure [31], secreted toxins are likely translocated into the EV lumen or associated with the EV membrane during this process (Figure 1).

Production of outer-membrane vesicles (OMVs) has been demonstrated to be a stress response characteristic of Gram-negative bacteria [32,33,34,35], and OMV production can contribute to bacterial survival in a hostile host environment [33,36,37,38]. As an opportunistic pathogen, *S. aureus* has also evolved adaptive mechanisms to survive stresses encountered in the environment and within the host during infection, such as iron limitation, oxidative stress, and exposure to antimicrobial agents [39,40,41,42,43,44]. Bacterial mechanisms to cope with these stressors include the regulated expression of virulence genes that may aid bacterial survival.

In this study, we describe the toxin content and the biological activities of *S. aureus* EVs, which represent a novel secretory system to deliver bacterial virulence determinants to human cells. We provide experimental evidence to show that the generation of *S. aureus* EVs is influenced by various environmental stresses, and those encountered in vivo, such as iron limitation and oxidative stress, enhance EV production and may contribute to the pathogenesis of staphylococcal infections.

## 2. Results

### 2.1. Purification of S. aureus EVs

**Figure 2:**
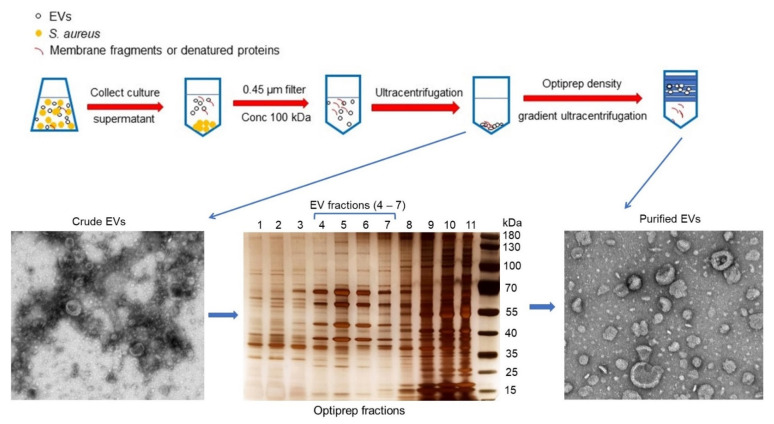
Purification of EVs from *S. aureus* cultures harvested at the post-exponential growth phase. Culture supernatants were filtered and concentrated 25-fold with a 100-kDa tangential flow filtration system, and the EVs were pelleted by ultracentrifugation. To remove non-membranous proteins, protein aggregates, and other contaminants, the EV sample was further purified by Optiprep density gradient ultracentrifugation. Gradient fractions were subjected to SDS-PAGE and silver stained. Fractions (#4–7) enriched for EVs were pooled, concentrated by diafiltration with phosphate buffered saline (PBS), and filtered. The purified EVs were negatively stained and examined by transmission electron microscopy.

We purified EVs from the culture supernatants of *S. aureus* JE2 [24], a USA300 strain that represents the major community-acquired methicillin-resistant clone in the United States. The EVs were harvested by concentrating filter-sterilized culture supernatants to remove molecules <100 kDa (Figure 2). Following ultracentrifugation, the pelleted crude EVs were purified from protein aggregates, membrane fragments, and other debris by Optiprep-based density gradient ultracentrifugation. Aliquots of Optiprep fractions were subjected to SDS-PAGE following by silver staining. Samples with similar protein banding patterns were pooled, subjected to diafiltration, and examined by transmission electron microscopy. EVs were distributed in fractions containing 20−35% Optiprep (Figure 2).

### 2.2. Toxin and Protease Components of S. aureus EVs

A proteomic analysis of purified JE2 EVs revealed a cargo comprised of 180 proteins [27], including an array of PFTs, proteases, and the superantigen SEIX. Table 1 summarizes our results, as well as data from groups who analyzed the protein content of EVs recovered from other *S. aureus* isolates [20,21,23,25,28,29]. In addition to differences in toxin cargo in EVs generated from different strains, Askarian et al. [23] reported differences in EV cargo when the same strain was grown in different nutrient media (Luria-Bertani vs. Brain Heart Infusion).

Alpha-hemolysin (Hla), the best-characterized PFT component of EVs [23,24,25,27,29], is secreted as a soluble monomer to form a pore by oligomerizing into heptamers on the host cell membrane [45]. Hla intoxicates many cell types, including erythrocytes, epithelial cells, endothelial cells, and innate immune cells, including neutrophils, monocytes, and macrophages [46]. The bi-component PFTs include the leukocidins LukSF-PV, LukED, HlgAB, HlgCB, and LukAB [47]. The leukocidins are commonly reported in EVs generated by a variety of *S. aureus* strains [20,21,23,28] (Table 1). Many hemopoietic cells, including monocytes, macrophages, neutrophils, erythrocytes, dendritic cells, and T cells, are targets of the leukocidins [47]. By affecting host cells that contribute to bacterial clearance, this toxin family plays a critical role in immune evasion by *S. aureus*.

PSMα, PSMβ, and delta hemolysin, detected in most *S. aureus* EVs [20,23,24,27,28] (Table 1), are small amphipathic α-helical peptides that lyse erythrocytes, neutrophils, monocytes, and epithelial cells in a receptor-independent fashion [10]. In addition to their cytotoxic activity toward eukaryotic cells, we showed that PSMα peptides promote *S. aureus* EV production by disrupting the integrity of the bacterial cytoplasmic membrane [24].

More than 20 staphylococcal enterotoxin (SEs) and staphylococcal enterotoxin-like (SEls) antigens have been identified from different *S. aureus* strains [11], and many of these have properties of superantigens. The incorporation of these enterotoxins within EVs is variable (Table 1). *S. aureus* JE2 produces SEK, SEQ, and SElX, but only SElX was detected in purified EVs [27]. Likewise, strain MW2 produces multiple SEs [48], such as SEA, SEC, SEG, SEH, SEK, SEL, and SElX, but none of these SEs were associated with its EVs [28]. *S. aureus* MSSA476 EVs carry SEA, SEK, and SEQ, but not SEC or SEH (encoded by the genome) [23]. Mastitis isolates N305 and RF122 (bovine) and O11 and O46 (ovine) produce various SEs and SEls, but none of these superantigens were identified in EVs purified from these staphylococcal strains [28].

Various *S. aureus* isolates produce exfoliative toxins (ETs) with serine protease activity, and expression of these toxins in the superficial layers of the skin results in staphylococcal scalded skin syndrome [49]. *S. aureus* strains JE2 and M060 produce ETA, but it was identified only in M060 EVs [20]. As shown in Table 1, ETC was detected in EVs purified from *S. aureus* strains M060, 03ST17, and 06ST1048 [20].

*S. aureus* isolates may produce up to 10 proteolytic enzymes, including serine proteases, serine protease-like (Spl) proteins, cysteine proteases, and one metalloprotease, and these enzymes modulate the stability of *S. aureus* proteins and target host molecules for degradation [7]. SplB and SplF, as well as cysteine proteases staphopain A and staphopain B, are associated with *S. aureus* JE2 EVs, but other proteases, including aureolysin, SplA, SplC, SplD, and SplE, are produced by JE2 but were not detected in EVs [27]. Protease cargo was also reported in EVs purified from other *S. aureus* strains, including MSSA476, ATCC 14458, and M060 [20,21,23] (Table 1).

As noted above, toxin and protease cargo in EVs isolated from various *S. aureus* isolates varies considerably and does not always correlate with bacterial strain toxin secretion. These data suggest a sorting mechanism that occurs during EV protein packaging, but details of that process and the mechanisms by which it occurs remain to be elucidated.

### 2.3. The Biological Activities of EV-Associated Toxins

**Figure 3:**
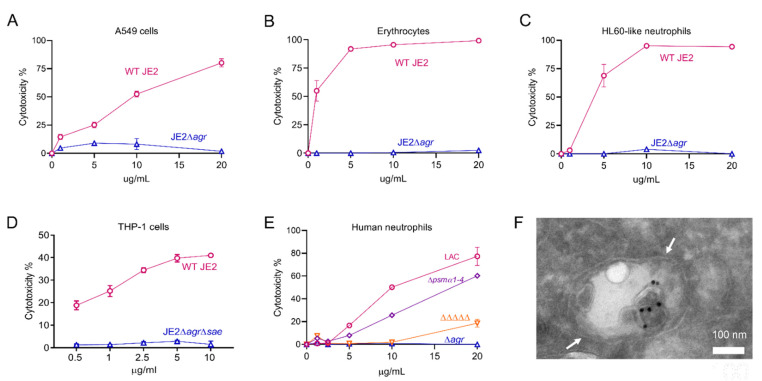
Cytotoxicity and cellular entry of *S. aureus* EVs. (**A**) Human lung A549 lung epithelial cells, (**B**) rabbit erythrocytes, (**C**) neutrophil-like HL60 cells, (**D**) THP-1 macrophages or (**E**) human neutrophils were incubated with increasing concentrations of EVs produced by indicated *S. aureus* WT or mutant strains, and cytotoxicity was evaluated. Each sample was tested in duplicate, and two independent experiments were performed with similar results. A representative experiment is shown. (**F**) Differentiated THP-1 macrophages were incubated with *S. aureus* EVs for 30 min. A representative electron micrograph shows antibody-labeled EVs (black dots) internalized within a macrophage endosomal compartment, denoted by white arrows.

The expression of many of the *S. aureus* PFTs and secreted enzymes is regulated by products of the accessory gene regulator (*agr*) operon [50]. The relative toxicity of EVs prepared from wild-type (WT) strain JE2 and JE2∆*agr* was assessed by incubating EVs in vitro with different cell types. JE2 EVs were toxic for A549 human epithelial cells at doses as low as 1 µg/mL, whereas EVs from JE2∆*agr* exhibited negligible toxicity (Figure 3A). Consistent with the fact that rabbit erythrocytes are susceptible to Hla, PSMs, and the leukocidins HlgAB and LukED [51,52,53], JE2 EVs exhibited hemolytic activity, whereas treatment with EVs from JE2∆*agr* resulted in no hemolysis (Figure 3B). HL60 neutrophils are susceptible to cytolysis induced by *S. aureus* leukocidins (including HlgAB, HlgCB, PVL-SF, LukED, and LukAB) and PSMs. As predicted, JE2 EVs, but not EVs from JE2∆*agr*, were cytolytic for HL60 cells (Figure 3C). Similarly, human macrophages are susceptible to Hla, PSMs, and leukocidins (including PVL-SF, LukED, LukAB, HlgAB, and HlgCB). JE2 EVs were toxic for differentiated THP-1 macrophages, whereas EVs from JE2∆*agr*∆*sae* showed negligible cytotoxicity (Figure 3D).

Alpha, but not beta, PSMs were detected in EVs produced by the USA300 strain JE2 [24,27]. To assess the relative cytolytic activities of EV-associated leukocidins versus alpha PSMs, we incubated human neutrophils with EVs purified from strain LAC, a mutant lacking either alpha PSMs (∆*psmα1–4*), or a mutant lacking all five leukocidins and Hla (denoted as ΔΔΔΔΔ). As shown in Figure 3E, EVs purified from the ∆*agr* mutant and the ΔΔΔΔΔ mutant exhibited negligible cytotoxicity. In contrast, EVs purified from the LAC∆*psmα1–4* mutant showed only a modest decrease in cytolytic activity compared to that of WT LAC EVs (Figure 3E). These findings indicate that EV-associated cytolysins are biologically active, and that the PFT cargo is largely responsible for the cytolytic activity of *S. aureus* EVs against mammalian host cells.

Utilizing confocal microscopy, we recently reported that fluorescent *S. aureus* EVs incubated with human macrophages were internalized within 30 to 60 min. This process was blocked when the cells were pretreated with dynasore, an inhibitor of dynamin-dependent endocytosis [27]. To expand upon these findings, differentiated THP-1 macrophages were incubated with purified *S. aureus* EVs, and the internalized EVs were visualized by transmission electron microscopy. As shown in Figure 3F, antibody-labeled EVs could be seen within macrophage endosomes within 30 min. Following internalization, *S. aureus* EVs induce macrophage pyroptosis and the release of cytokines IL-1β and IL-18 through the activation of NLRP3 inflammasomes [27].

To assess whether internalization was essential for EV cytotoxicity, THP-1 macrophages were pretreated with dynasore or the DMSO vehicle control before incubation with EVs, and cytotoxicity was determined by measuring lactate dehydrogenase (LDH) release. Blockage of EV internalization by dynasore did not reduce cytotoxicity in comparison to the DMSO control (Figure S1). Because cellular entry was not essential for EV-induced cytotoxicity, these data suggest that biologically active PFTs associated with the surface of *S. aureus* EVs [27] were able to bind to their cognate receptors on human macrophages to effect membrane damage.

### 2.4. Effects of Stress on S. aureus EV Production

To survive in a hostile host environment, *S. aureus* has developed adaptive mechanisms to respond to environmental changes and stressors encountered during infection [54,55,56]. Although increasing evidence suggests that EVs play a role in the pathogenesis of staphylococcal infections, there is scarce information available on the environmental conditions that influence the generation of EVs. To this end, we evaluated the in vitro production of *S. aureus* EVs under different physiological stresses.

#### 2.4.1. Effect of Temperature on EV Production

**Figure 4:**
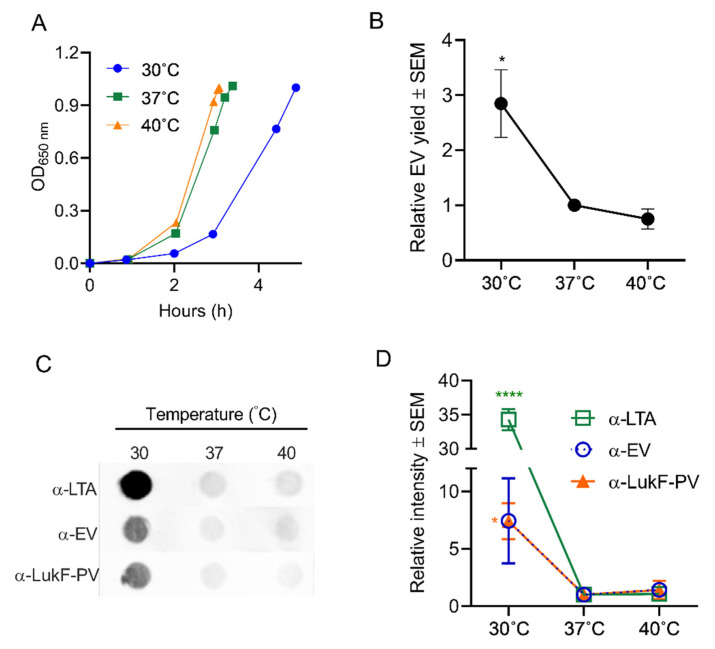
Effect of temperature on EV production. (**A**) *S. aureus* was cultivated in 100 mL tryptic soy both (TSB) at the indicated temperatures until an OD of 1 was achieved. (**B**) EV production quantified by relative protein yield (compared to 37 °C cultures) or (**C**) by reactivity of dot immunoblots of EV suspensions with indicated antibodies. Dot bots were performed at least three times, and a representative image is presented. (**D**) Mean relative intensity values (compared to 37 °C cultures) pooled from 3 independent immunoblot experiments are shown. One-way ANOVA with Dunnett’s multiple comparison tests were used for statistical analyses of protein yield and relative intensity of dot blot images. * *p* < 0.05, **** *p* < 0.0001.

*S. aureus* may encounter shifts in temperature as it transits from the anterior nares, the skin, or from inanimate objects to deeper tissues during the establishment of infection. Similarly, higher temperatures associated with fever may be encountered by the bacterium during an acute infection. To determine whether these temperature shifts affect EV production, *S. aureus* JE2 was cultivated to an OD of 1 at either 30 °C, 37 °C, or 40 °C. As shown in Figure 4A, the *S. aureus* doubling time during growth at 30 °C was longer than that of cultures grown at 37 °C or 40 °C. A quantitative analysis of EV protein yield showed that significantly more EVs were recovered from the 30 °C cultures than from cultures grown at the higher temperatures (Figure 4B). Relative EV production was further evaluated by quantitative dot immunoblots with antibodies against EVs, lipoteichoic acid (LTA), or Panton-Valentine leukocidin F subunit (LukF-PV), since these antigens are abundant within JE2 EVs [24,27]. As shown in Figure 4C,D, all three antibodies reacted more strongly with EVs generated from 30 °C cultures compared to EVs from cultures incubated at 37 °C or 40 °C. The reason for the enhanced reactivity of LTA antibodies against EVs from 30 °C cultures remains unclear. Whereas LTA is essential for *S. aureus* growth at 37 °C, it is not essential for growth at 30 °C [57]. Whether LTA synthesis is upregulated at the lower temperature or whether EVs selectively incorporate more of this anionic polymer into its membranes at 30 °C remains to be determined. The specificity of the dot immunoblot assay was tested by incubating serial dilutions of EVs generated at the different temperatures with antiserum raised to *S. aureus* EVs or with preimmune mouse serum. As expected, EVs were reactive with EV antiserum, but not with preimmune mouse serum (Figure S2).

#### 2.4.2. Effects of Oxidative Stress on EV Production

*S. aureus* encounters oxidative stress during aerobic respiration when incomplete reduction of oxygen leads to the generation of reactive oxygen species, such as superoxide anions (O^−2^) and hydrogen peroxide (H_2_O_2_) [42]. *S. aureus* may also encounter oxidative stress as a result of interactions with host phagocytes that produce NADPH oxidase and generate O^−2^ during the oxidative burst. O^−2^ can undergo dismutation to H_2_O_2_ that can be used by the myeloperoxidase complex to produce the bactericidal compound hypochlorite. Ciprofloxacin and other bactericidal antibiotics have also been shown to increase oxidative stress in bacterial cells by increasing the production of reactive oxygen species [58].

**Figure 5:**
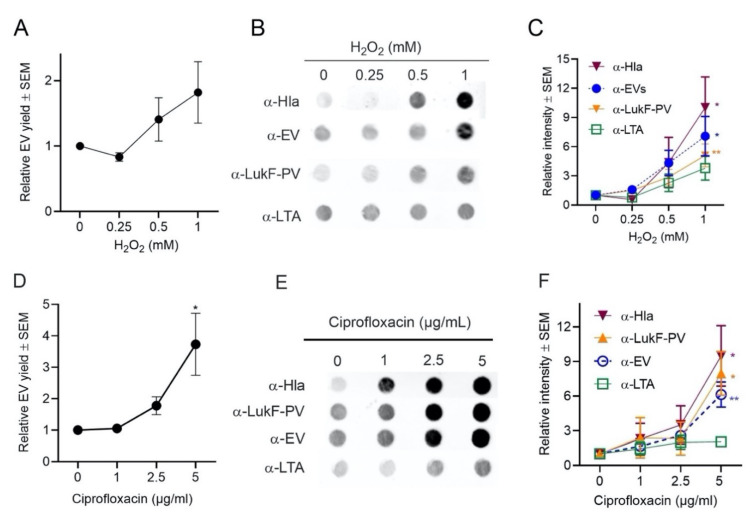
EV production was enhanced by oxidative stress. (**A**) EV production from *S. aureus* grown in TSB supplemented with indicated concentrations of H_2_O_2_ was evaluated by quantification of relative EV protein yield or (**B**) by dot blots of EV suspensions probed with indicated antibodies. (**C**) Relative intensity of dot blot images pooled from 3 to 4 independent experiments are shown. (**D**) EV production by *S. aureus* grown in TSB supplemented with indicated concentrations of ciprofloxacin was evaluated by quantification of relative EV protein yield or (**E**) by dot immunoblots of EV suspensions probed with indicated antibodies. (**F**) Relative intensity of dot blot images pooled from 3 to 4 independent experiments are shown. EV protein yield was determined from at least three independent experiments. One-way ANOVA with Dunnett’s multiple comparison tests were used for statistical analysis of protein yield and relative intensity of dot blot images. * *p* < 0.05, ** *p* < 0.01.

To evaluate whether oxidative stress affects the production of EVs, strain JE2 was cultivated in TSB, and H_2_O_2_ was added to log-phase cultures at concentrations ranging from 0 to 1 mM. To counteract peroxide degradation, fresh H_2_O_2_ was added at four intervals until the culture reached an OD of 1.0. *S. aureus* growth rates were minimally affected by the subinhibitory H_2_O_2_ concentrations that were used in our studies (Figure S3A). Protein quantification showed a dose-dependent increase in relative EV production when *S. aureus* was grown in TSB supplemented with H_2_O_2_ (Figure 5A). Likewise, a dose-dependent increase in EV production was observed when EVs were probed by immunoblot with antibodies against *S. aureus* EVs, LukF-PV, LTA, or Hla (Figure 5B,C).

Similarly, when *S. aureus* was grown in subinhibitory concentrations of ciprofloxacin (Figure S3B), protein quantification assays (Figure 5D) and dot immunoblot analyses (Figure 5E) showed that the production of EVs was increased compared to cultures lacking ciprofloxacin. EV immunoblots probed with antibodies to Hla, LukF-PV, or EVs showed a dose-dependent increase in signal, but there was only a minimal increase when immunoblots were probed with LTA antibodies (Figure 5F). Lotz et al. reported that treatment with a low concentration of ciprofloxacin resulted in LTA release by *S. aureus* cells [59]. Whether this accounts for the minimal signal observed when EVs were probed with LTA antibodies merits further investigation. In contrast, protein quantification assays and dot blot analyses showed that the production of EVs was decreased when *S. aureus* JE2 was grown in a subinhibitory concentration (0.05 µg/mL) of erythromycin (Figure S4), a bacteriostatic antibiotic that inhibits protein synthesis.

#### 2.4.3. Effect of Iron Limitation on *S. aureus* EV Production

Iron is an essential nutrient for both human and microbes and serves as a cofactor in many biological processes. The host tightly regulates iron distribution within the body, and this serves as an innate immune mechanism against invading microbes. Iron in humans is sequestered intracellularly, complexed within hemoglobin inside erythrocytes. Free iron is tightly bound by transferrin, lactoferrin, and haptoglobin [60,61]. Bacterial production of vesicles by *Haemophilus influenzae* and *Mycobacterium tuberculosis* [62,63] was enhanced under iron-limiting conditions, and the latter produced EVs containing mycobactin, which supports the replication of iron-starved mycobacteria [62]. To investigate whether iron limitation influenced the generation of *S. aureus* EVs, we cultivated strain JE2 in TSB with the iron chelator 2,2-dipyridyl (DIP) [64], at concentrations (0 to 0.4 mM) that did not impact bacterial growth (Figure S3C).

**Figure 6:**
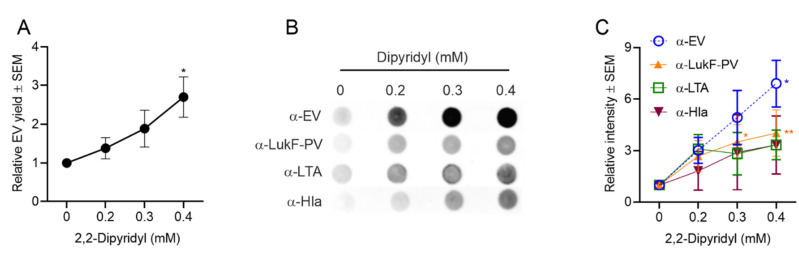
*S. aureus* EV production was enhanced in iron-depleted media as determined by (**A**) quantification of relative EV protein yield and (**B**) dot immunoblots of EV suspensions with indicated antibodies. (**C**) Relative intensity of dot blot images was assessed from 3 to 4 independent experiments, and a representative dot blot is shown. EV protein yield was analyzed from at least three independent experiments. One-way ANOVA with Dunnett’s multiple comparison tests were used for statistical analysis of protein yield and relative intensity of dot blot images. * *p* < 0.05, ** *p* < 0.01.

Protein quantification showed that *S. aureus* EV yield was enhanced in a dose-dependent fashion with increased concentrations of DIP in the medium (Figure 6A). Likewise, dot blot analyses of EV preparations with antibodies to EVs or LukF-PV showed a significant increase in signal for samples cultivated with 0.4 mM DIP. EV reactivity with antibodies to LTA and Hla were enhanced up to threefold in iron-deficient media, but these differences did not reach significance (Figure 6B,C). It is possible that more EVs are generated in iron-limited media, but that the EV cargo is modified under these conditions. Future experiments will address these findings.

#### 2.4.4. Effect of Osmotic Stress on *S. aureus* EV Production

**Figure 7:**
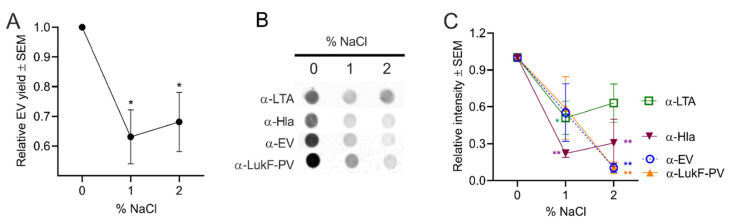
*S. aureus* EV production was reduced by osmotic stress. (**A**) EV production from *S. aureus* grown in TSB supplemented with 1 to 2% NaCl was evaluated by quantification of relative EV protein yield or (**B**) by dot immunoblots of EV suspensions probed with indicated antibodies. (**C**) Relative intensity of dot blot images pooled from three independent experiments are shown, and a representative blot is shown. EV protein yield was analyzed from three independent experiments. One-way ANOVA with Dunnett’s multiple comparison tests were used for statistical analysis of protein yield and relative intensity of dot blot images. *p < 0.05, ** *p* < 0.01.

The presence of high salt in the environment, food, or on human skin can generate osmotic stress on *S. aureus* cells, resulting in water loss, low turgor pressure, and cell shrinkage [56]. To evaluate the effect of osmotic stress on EV production, *S. aureus* was grown in TSB medium supplemented with 1% or 2% NaCl. Although the added salt only showed a minimal effect on *S. aureus* growth (Figure S3D), EV protein yield was significantly reduced under high salt conditions (Figure 7A). Likewise, dot immunoblot analysis of EV preparations with antibodies to EVs, LukF-PV, or Hla showed reduced signals in samples cultivated in TSB + NaCl (Figure 7B,C). The reduction in signal was less apparent with antibodies to LTA (Figure 7C), suggesting that the LTA content of EVs was somewhat enriched compared to that of the other antigens. Kho and Meredith reported that the LTA component of the cell wall has been shown to be important in withstanding salt-induced osmotic stress [65].

#### 2.4.5. Effect of Ethanol Treatment on *S. aureus* EV Production

**Figure 8:**
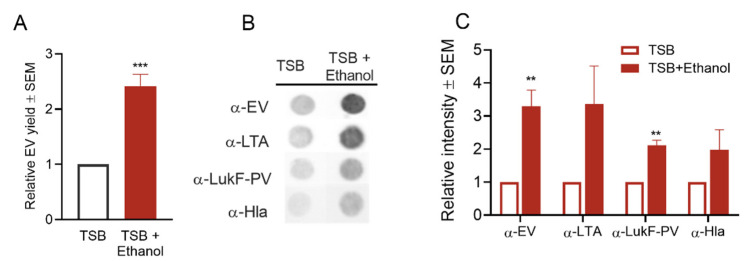
*S. aureus* EV production was enhanced by ethanol stress. (**A**) Quantification of relative EV protein yield and (**B**) dot blots of EV suspensions probed with indicated antibodies showed increases in EV production from cultures with added ethanol. (**C**) The relative intensity of dot blot images pooled from 3 independent experiments was calculated, and a representative image is shown in (**B**). EV protein yield was calculated from three independent experiments. ** *p* < 0.01, *** *p* < 0.001, as determined by the Student *t*-test.

Ethanol, widely used in the disinfection of skin and medical devices in the healthcare environment, causes disruption of membrane structures [66] and interferes with cell division [67]. Although high concentrations of ethanol are used in disinfection, residual ethanol left on surfaces may be low due to evaporation or dilution. Low concentrations of ethanol can exert envelope stress on *S. aureus,* as shown by altered gene expression profiles [41]. To address the effect of ethanol on EV production, we cultivated *S. aureus* in TSB + 1% ethanol. Although the bacterial growth rate was not affected (Figure S3E), EV yield was significantly enhanced in the presence of ethanol (Figure 8A). Similarly, dot blot analysis of EV preparations with antibodies to EVs, LTA, LukF, and Hla showed increased signals (Figure 8B,C), confirming the positive effect of ethanol stress on EV generation.

## 3. Discussion

*S. aureus* EV formation is common among *S. aureus* strains, including antibiotic-resistant isolates, and much information about their biological activities has been revealed during the past decade [19,20,21,22,23,24]. Staphylococcal EVs package a diverse array of components, including cytosolic, surface, and membrane proteins, as well as surface adhesins, lipoproteins, and toxins [19,20,21,24]. Staphylococcal products secreted as soluble molecules in vivo can interact with host cells, but they may be subject to destruction by proteases or antibody neutralization. Because *S. aureus* EVs can be endocytosed within host cells [27], EVs may serve as a novel secretory system for *S. aureus* to effectively transport toxins and other EV cargo to intracellular compartments. The fate of EV cargo upon internalization, however, still remains to be elucidated.

The cytolytic activity of *S. aureus* EVs is largely dependent upon its PFT cargo, indicating that EV-associated toxins are biologically active. We reported that EVs were internalized within human macrophages via a dynamin-dependent endocytic pathway [27], and electron micrographs revealed internalized *S. aureus* EVs within a macrophage endosome-like structure (Figure 2). Whether intracellular EV-associated toxins exert a cytolytic effect on host cells is still unclear. Previous studies demonstrated that PFTs and PSMs are active intracellularly and can facilitate the escape of *S. aureus* from subcellular compartments of host cells [68,69,70,71,72,73,74] and trigger intracellular signaling cascades [69,70,72]. Here we show that blockage of dynamin-dependent endocytosis did not abrogate the cytotoxicity of *S. aureus* EVs. It is likely that certain EV-associated leukocidins [27], such as LukAB, bind to their host receptors localized at cell surface before EV internalization, resulting in cytotoxicity.

Inflammasome activation is important in controlling staphylococcal infections, particularly in mounting an effective host innate immune response [75,76,77]. However, unregulated inflammasome activation may result in an exaggerated inflammatory response that leads to host tissue damage, particularly in the lung [78,79]. *S. aureus* culture supernatants, containing lipoproteins and secreted PFTs, activate inflammasomes by providing both the priming and second stimulus [80]. Because most lipoproteins are EV-associated [81], it is likely that EVs with their toxic cargo may contribute to inflammasome activation during infection. Hong et al. showed that EV-associated Hla was more cytotoxic to HaCaT keratinocytes than free Hla, and that Hla-negative EVs did not induce keratinocyte death [25]. Moreover, EV-associated Hla, but not soluble Hla, caused atopic dermatitis-like dermal inflammation in mice [25,82]. These studies underscore the potential effects that EV-associated PFTs may exert on *S. aureus* infections.

The mechanisms whereby toxins and other proteins are packaged within EVs secreted from the cytoplasmic membrane of Gram-positive bacteria are unknown. We reported that staphylococcal lipoproteins may be involved in protein sorting of EV cargo since their presence modulated EV biogenesis, including its toxin cargo [27]. Recently, a proteomic analysis of EVs from different *S. aureus* strains originating from human, bovine, and ovine hosts indicated that a core EV proteome was shared by EVs secreted from all tested strains [28], suggesting the existence of a selective mechanism for EV cargo sorting. The same group also found that EV-associated proteins were more positively charged at a physiological pH. They postulated that positively charged proteins were recruited to the site of EV formation by negatively charged microdomains at the cytoplasmic surface of the membrane through electrostatic interactions [28], a process that can direct the subcellular localization of proteins [83]. Additional studies are clearly needed to better understand EV biogenesis and the selective packaging of EV cargo.

Although production of EVs has been characterized in various *S. aureus* strains under optimal growth conditions, little is known about EV production in the environment or during infection. Unlike bacteria cultivated under optimal laboratory conditions, bacterial cells persisting in the environment or infecting a host can encounter many stressors, and such conditions may influence EV production. High osmotic conditions result in a thicker bacterial cell wall [84], forming a barrier for EV release, and this finding was consistent with our observation that *S. aureus* EV production was significantly reduced under high osmotic conditions. Alterations in staphylococcal membrane phospholipid content have also been observed in response to changes in salinity [56]. *S. aureus* grown at 30 °C, a temperature encountered during colonization of the skin or nares, produced a greater EV yield than cultures maintained at 37 °C or 40 °C. The cell membrane of *S. aureus* cultivated at 37 °C is mainly composed of straight-chain and branched-chain saturated fatty acids, whereas at lower temperatures staphylococci modify the composition of their membrane to comprise mainly unsaturated fatty acids, resulting in higher membrane fluidity [56,85]. This may increase membrane curvature, resulting in increased vesiculation. However, *S. aureus* grown at 30 °C also showed a slower growth rate in vitro, and thus it is possible that more EVs were produced simply because of the longer growth period required to reach an OD of 1. In our subsequent experiments, we were careful to choose concentrations of stressors that did not significantly affect the staphylococcal growth rate.

We showed in a previous study that *S. aureus* EV yield was significantly increased by culturing the bacteria in media containing subinhibitory concentrations of penicillin G, and that enhanced EV production correlated with decreased peptidoglycan cross-linking [23]. In this study, we show that EV production was diminished when *S. aureus* was cultivated in subinhibitory concentrations of erythromycin, a bacteriostatic antibiotic that inhibits protein synthesis. In contrast, EV yield increased when *S. aureus* was grown in TSB with subinhibitory concentrations of the bactericidal antibiotic ciprofloxacin, a fluoroquinolone that targets DNA gyrase and topoisomerase and exerts oxidative stress in treated bacteria [86,87]. Oxidative stress was further explored by adding subinhibitory concentration of H_2_O_2_ during the exponential phase of staphylococcal growth, resulting in the generation of more EVs than untreated cultures. *S. aureus* adapts to oxidative stress by producing carotenoid pigments like staphyloxanthin that maintain the integrity of the cell membrane [42]. The bacterium also produces detoxifying enzymes, such as superoxide dismutase to break down superoxide, catalase to degrade H_2_O_2_, and peroxiredoxins such as AhpC to detoxify alkyl hydroperoxides by converting them to their corresponding alcohols [42]. Because all three of these detoxifying enzymes are included within *S. aureus* EV cargo [24,27], it is possible that increased EV yield may represent an adaptive bacterial survival mechanism in the oxidative environment encountered during infection.

Our experimental results revealed that EV yield was enhanced when *S. aureus* was cultivated in iron-depleted medium. Iron is an essential nutrient for all living organisms, but there is limited free iron for bacterial utilization during infection. *S. aureus* EV components Hla, LukED, and HlgAB lyse erythrocytes to release hemoglobin and heme [44], thus promoting bacterial iron acquisition. Because they can serve as a vehicle to transport PFTs to the environment and host cells, EVs may play a role in iron acquisition during infection. The *S. aureus psma* genes, also highly expressed under conditions of iron depletion [88,89], encode peptides with surfactant-like activity. These PSMs promote EV production by enhancing membrane curvature, partially disrupting the cytoplasmic membrane and enhancing EV formation and yield [24].

## 4. Conclusions

The cargo of *S. aureus* EVs includes multiple exoproteins, including toxins and proteases, cytoplasmic proteins, adhesins, and lipoproteins. *S. aureus* EVs, but not EVs from PFT-deficient strains, were cytolytic to a variety of mammalian cell types, and EV internalization was not essential for cytotoxicity. EV production may serve as a virulence mechanism for *S. aureus* to transport toxins and other components of its secretome into host cells, while protecting the contents of the EV lumen from degradation or neutralization. We demonstrate that production of EVs is modulated by environmental stressors, such as antibiotics, oxidative stresses, and iron depletion, suggesting that EV generation may represent an adaptive mechanism for *S. aureus* growth in a hostile host environment. Temperature, ethanol, and salt also modulated EV production, confirming the impact of stress on EV biogenesis. Additional comprehensive studies are needed to understand the process of *S. aureus* EV cargo sorting, biogenesis, and the relevance of EVs to the pathogenesis of staphylococcal disease.

## 5. Materials and Methods

### 5.1. Purification and Proteomic Analysis of EVs

EVs were purified from *S. aureus* JE2, LAC, LAC ∆*psmα1-4*), and LAC ΔΔΔΔΔ [90] as described [24]. Briefly, *S. aureus* strains were cultivated with shaking in TSB to an OD of 1.2. The culture supernatants were filtered and concentrated 25-fold with a 100-kDa tangential flow filtration system (Centramate, Pall Corp., Hauppauge, NY, USA). EVs were pelleted from the retentate by ultracentrifugation at 150,000× *g* for 3 h at 4 °C. To remove non-membranous proteins, protein aggregates, and other contaminants, EV samples were overlaid by gradient layers of Optiprep medium ranging from 40% to 15%. After centrifugation for 16 h, aliquots of 1 mL fractions were subjected to SDS-PAGE and silver stained. Fractions enriched for EVs were pooled and concentrated by diafiltration with phosphate buffered saline (PBS). Purified EVs were filtered again and stored at −80 °C. Proteomic analyses of EVs by liquid chromatography–tandem mass spectrometry (LC–MS/MS) were reported previously [24,27].

### 5.2. Transmission Electron Microscopy

Purified *S. aureus* EVs were visualized by TEM as described [24]. For immunogold labeling of intracellular EVs, differentiated THP-1 cells were incubated with JE2 EVs for 30 min before the cells were washed with PBS and pelleted by centrifugation at 3000× *g*. The pelleted cells were fixed in 4% paraformaldehyde for 2 h at room temperature, and then placed in PBS containing 0.2 M glycine to quench free aldehyde groups. The fixed cell pellets were cryoprotected by incubating with PBS containing 2.3 M sucrose, frozen in liquid nitrogen, and sectioned on an ultramicrotome at −120 °C. The sections were transferred to formvar/carbon coated copper grids and stained with antibodies to *S. aureus* EVs, followed by a protein A-gold (5 nm) conjugate. The grids were counterstained and embedded by incubation with 0.3% uranyl acetate in 2% methyl cellulose. The samples were imaged on a JEOL1200EX electron microscope (JEOL, Peabody, MA, USA) equipped with an AMT 2k CCD camera (Advanced Microscopy Techniques Corp., Danvers, MA, USA).

### 5.3. Cell Culture and EV Cytotoxicity

THP-1 cells (ATCC, Manassas, VA, USA) were maintained and differentiated as described [27]. The human A549 lung epithelial cells were maintained in RPMI-1640 (Gibco, Grand Island, NY, USA) supplemented with 10% (vol/vol) fetal bovine serum (FBS; Hyclone, Logan, UT, USA), 50 U/mL penicillin, and 50 μg/mL streptomycin. Human neutrophils, isolated from the blood of healthy donors with Polymorphprep (Cosmo Bio USA, Inc., Carlsbad, CA, USA), were washed and suspended in RPMI-1640 containing 5% FBS (Hyclone, Logan, UT, USA). HL60 cells were maintained and differentiated as we described previously [91].

The relative cytotoxicity of EVs (1 to 20 µg/mL) toward different cell types was determined. A549 lung epithelial cells were incubated overnight in a 96-well plate at 37 °C with EVs. Cytotoxicity was assessed by measuring the LDH in culture supernatants with an LDH cytotoxicity assay kit (Thermo Fisher Scientific, Waltham, MA, USA). Differentiated HL60 cells or human neutrophils were seeded in 96-well plates and treated with EVs, 1% Triton X-100 lysis buffer, or LukSF-PVL (1 µg/mL; IBT Bioservices, Rockville, MD, USA) for 4 h at 37 °C. Cytotoxicity was measured with a CellTiter kit (Promega, Madison, WI, USA). A 2% rabbit erythrocyte suspension was mixed with EVs or the positive control Hla (1 µg/mL; IBT Bioservices, Rockville, MD, USA;) in a 96-well plate for 1 h at 37 °C. The erythrocytes were pelleted by centrifugation, and cytotoxicity was determined by measuring the OD_545 nm_ of the supernatant with an ELISA reader. Differentiated THP-1 macrophages grown in 48-well plates were incubated with EVs or lysis buffer for 4 h at 37 °C. Cytotoxicity was assessed by measuring the LDH level in culture supernatants using an LDH cytotoxicity assay kit.

### 5.4. Stress Treatments

To assess the effects of growth temperature on EV production, 100 µl of an overnight culture of *S. aureus* JE2 was used to inoculate 100 mL of prewarmed (30 °C, 37 °C, or 40 °C) TSB medium. The cultures were cultivated with shaking (200 rpm), and bacterial growth curves were generated by recording optical density (OD_650 nm_) readings until the post exponential phase of growth (OD_650_ 1.2). Culture supernatants were collected by centrifugation (10,000× *g*, 10 min) at 4 °C and filter sterilized with a 0.45 µm filter. After the supernatant was concentrated 4-fold with a 100-kDa tangential flow filtration system (Pall Corp., Hauppauge, NY, USA), the retentate was filtered again and centrifuged at 150,000× *g* for 3 h at 4 °C to pellet the vesicles. The EVs were resuspended in 250 µl sterile PBS and sterilized by passage through a 0.45 µm filter. EV protein concentrations, determined with a BioRad protein assay, were expressed relative to EVs harvested from 37 °C cultures. Immunoblot analyses on each sample were performed as described below.

To measure the effects of a variety of stressors on EV production, we inoculated 100 mL TSB supplemented with or without 1% ethanol, 1 or 2% NaCl, 0.2, 0.3, or 0.4 mM 2,2-dipyridyl, 1, 2.5, or 5 µg/mL ciprofloxacin, or 0.05 µg/mL erythromycin as described above. The cultures were incubated with shaking (200 rpm) at 37 °C to an OD_650_ of 1.2. Bacterial growth curves were generated with each culture to assess relative growth rates of treated and untreated samples. Culture supernatants were collected, concentrated by tangential flow filtration, and EVs were isolated as described above.

*S. aureus* cultures treated with H_2_O_2_ were inoculated as described above and cultivated at 37 °C until early log phase (OD_650_ ~0.2). Bacterial cells were pelleted (10,000× *g*, 10 min) at 4 °C and resuspended in an equal volume of fresh 37 °C TSB medium supplemented with final concentrations (0, 0.25, 0.5, or 1 mM) of H_2_O_2_. Fresh H_2_O_2_ was added to each culture at OD readings of 0.4, 0.6, 0.8 to counteract H_2_O_2_ degradation. Cultures were grown to an OD_650_ of 1 before collecting the culture supernatants for EV isolation and analyses.

### 5.5. Dot Immunoblot Analysis of EV Yield and Content

EVs from stressed culture conditions were isolated as described above; 100 µL of serial twofold dilutions of the EV samples were applied to nitrocellulose membranes using a 96-well Bio-dot apparatus (Bio-Rad, Hercules, CA, USA). To block the staphylococcal IgG binding proteins Spa and Sbi, the membranes were blocked for 1 h at room temperature with either rabbit or guinea pig IgG (2 µg/mL) in PBS + 5% skim milk. The membranes were washed with PBST (PBS + 0.05% Tween-20) and incubated overnight at 4 °C with one of four primary antibodies. These included sera (diluted 1:1000 in PBS + 0.1% Tween-20 and 5% skim milk) collected in a previous study [24] wherein mice were immunized with purified *S. aureus* EVs. Alternative the membranes were incubated with 1 µg/mL of Hla mAb 6C12, rabbit anti-LukF-PV, or LTA mAb (all obtained from IBT Bioservices, Rockville, MD, USA). After washes with PBST, the membranes were incubated at RT for 1 h with either HRP-conjugated goat anti-rabbit antibody or HRP-conjugated goat anti-mouse antibody (1:10,000 dilution in PBST + 5% skim milk). The membranes were washed with PBST, developed with a chemiluminescent HRP substrate (Thermo Fisher Scientific, Waltham, MA, USA), and imaged with an iBright FL1500 instrument (Thermo Fisher Scientific, Waltham, MA, USA). Signal intensity of dot blot mages (chosen at the same EV dilution for all samples subjected to a given stress) were quantified by Image J (National Institutes of Health, Bethesda, MD, USA). The chemiluminescence of treated samples was compared relative to untreated samples for each stress condition at the optimal EV dilution.

### 5.6. Statistical Analyses

Statistical analyses were performed using Prism 7.0 (GraphPad, San Diego, CA, USA). Statistical significance was calculated using one-way ANOVA with Dunnett’s multiple comparison test. Samples treated with or without ethanol or Em were analyzed by an unpaired Student *t*-test. A *p* value of ≤0.05 was considered significant.

## Figures and Tables

**Table 1:** Toxins and proteases associated with EVs from different *S. aureus* isolates.

Protein Family	EV-Associated Protein	EV Strain Source	References
Pore-forming toxins	Alpha hemolysin	JE2, 8325-4, ATCC 14458, MSSA476	[21,23,24,25,27,29]
	Leukocidin ED	JE2	[24,27]
	Leukocidin SF-PVL	JE2	[24,27]
	Leukocidin HlgAB	JE2, M060, 03ST17, 06ST1048, MSSA476	[20,23,24,27]
	Leukocidin HlgCB	JE2, ATCC 14458, 03ST17, 06ST1048, M060, MSSA476	[20,21,23,24,27]
	Leukocidin LukAB	JE2, N305, RF122, O11, O46, MW2, MSSA476	[23,24,27,28]
	Leukocidin LukMF’	RF122, O11, O46	[28]
	Delta hemolysin	JE2, MW2, N305, O11, O46, RF122, MSSA476, M060, 03ST17, 06ST1048	[20,23,24,27,28]
	Phenol soluble modulins (alpha)	JE2, MW2, N305, O11, O46, RF122, M060	[20,24,27,28]
	Phenol soluble modulins (beta)	N305, RF122, O11, O46, MW2, MSSA476	[23,28]
Superantigens	SEA, SEK	MSSA476	[23]
	SEQ	ATCC 14458, MSSA476	[21,23]
	Staphylococcal enterotoxin like-toxin X (SElX)	JE2	[24,27]
Exfoliative toxins	ETA	M060	[20]
	ETC	M060, 03ST17, 06WT1048	[20]
Proteases	Cysteine protease Staphopain A (ScpA)	JE2, ATCC 14458, M060, MSSA476	[20,21,23,24,27]
	Cysteine protease Staphopain B (SspB)	JE2, MSSA476	[23,24,27]
	Aureolysin	MSSA476	[23]
	SplB, SplF	JE2	[24,27]

## Data Availability

The data presented in this study are available on request from the corresponding author upon reasonable request. Mass spectrometry proteomics data were deposited in the ProteomeXchange Consortium (http://proteomecentral.proteomexchange.org/cgi/GetDataset) via the PRIDE partner repository with the data set identifier PXD014888 and PXD007953, respectively.

## Supplementary Materials

The following are available online at https://www.mdpi.com/2072-6651/13/2/75/s1, Figure S1: Blockage of dynamin-dependent endocytosis did not abrogate the cytotoxicity of *S. aureus* EVs toward differentiated THP-1 macrophages, Figure S2: Control dot immunoblot of EVs generated from cultures incubated at different temperatures, Figure S3: Growth curves of *S. aureus* JE2 in TSB medium in the presence or absence of indicated concentrations of (A) H_2_O_2_, (B) ciprofloxacin, (C) 2,2-dipyridyl, (D) NaCl, or (E) ethanol, Figure S4: The effect of erythromycin on *S. aureus* EV production.
