# Supplementary material for: Staphylococcus aureus Extracellular Vesicles: A Story of Toxicity and the Stress of 2020
Source: Toxins (Basel). 2021 Jan 20;13(2):75. doi: 10.3390/toxins13020075 (PMC7909408; doi:10.3390/toxins13020075)
Supplement: Supplementary file 1 [file toxins-13-00075-s001.pdf]

Supplementary Materials: *Staphylococcus aureus* Extracellular Vesicles: A Story of Toxicity and the Stress of 2020

Xiaogang Wang, Paul F. Koffi, Olivia F. English and Jean C. Lee

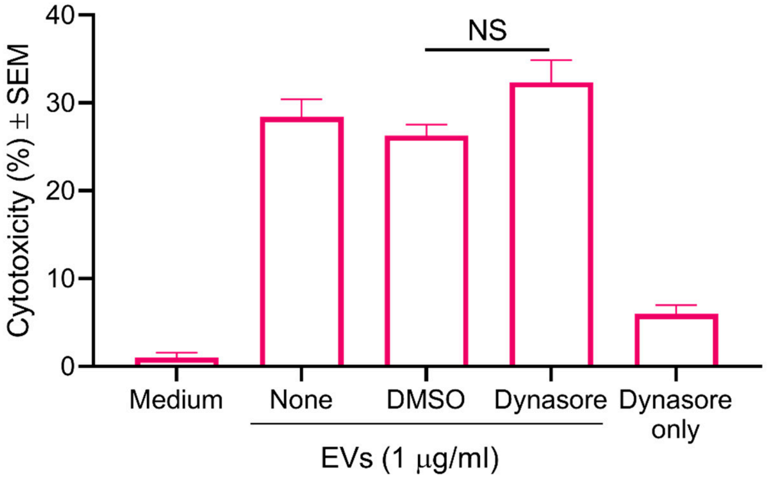

Figure S1. Blockage of dynamin-dependent endocytosis did not abrogate the cytotoxicity of *S. aureus* EVs toward differentiated THP-1 macrophages. Cytotoxicity was measured by release of LDH in culture supernatants of differentiated THP-1 cells that were treated with the dynamin inhibitor dynasore (40 μM) or DMSO for 1 h prior to incubation for 4 h with 1 μg/ml *S. aureus* EVs. A One-way ANOVA with Dunnett's multiple comparison test was used for statistical analysis. NS, Not significant.

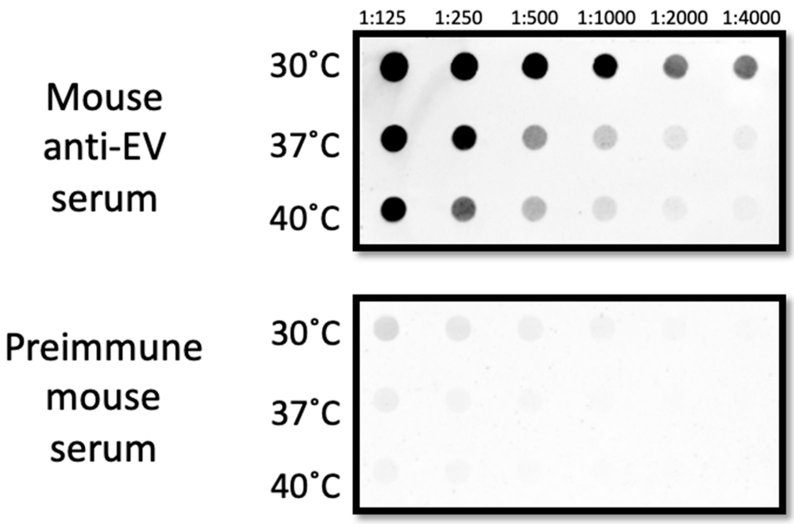

Figure S2. Control dot immunoblot of EVs generated from cultures incubated at different temperatures. *S. aureus* was cultivated in 100 ml TSB at the indicated temperatures until an OD of 1 was achieved. Two-fold serial dilutions of EV samples isolated from different cultures were probed with pooled serum from mice immunized with *S. aureus* EVs or preimmune normal mouse serum diluted 1:1000.

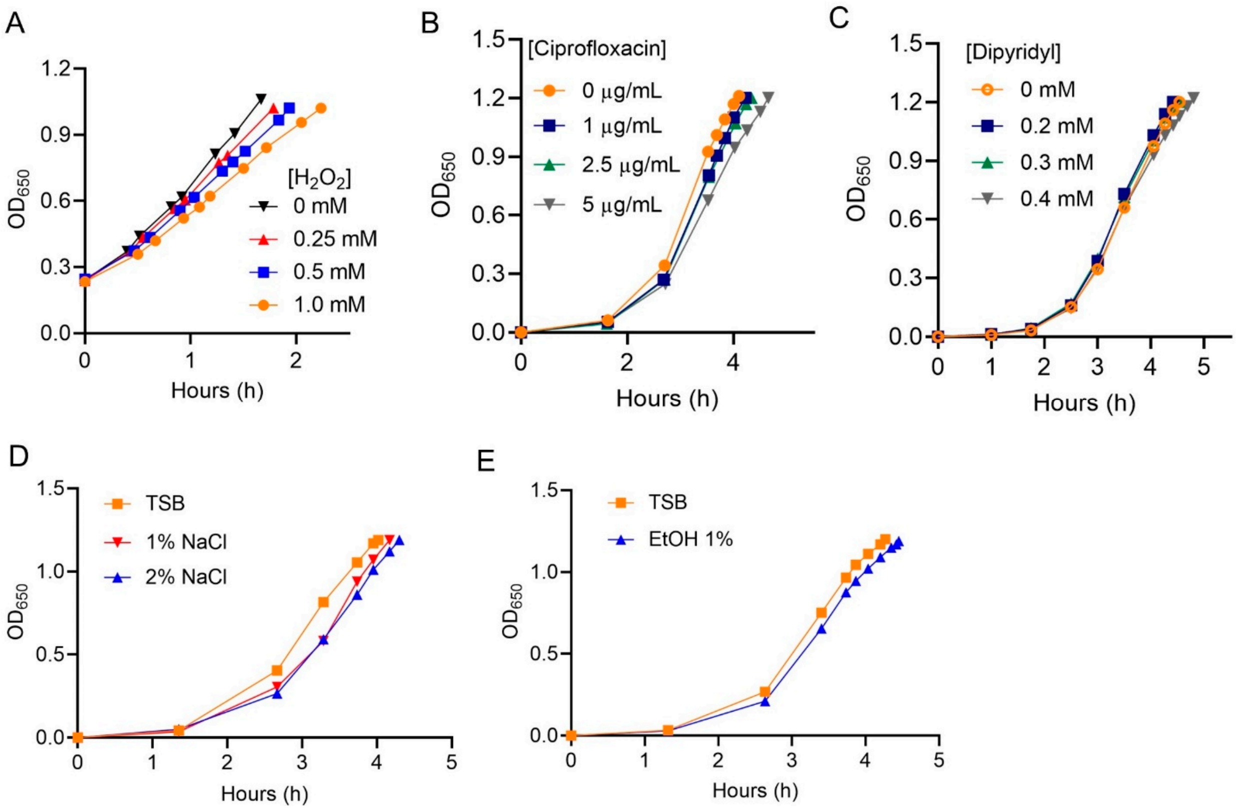

Figure S3. Growth curves of *S. aureus* JE2 in TSB medium in the presence or absence of indicated concentrations of (**A**) H_2O_2, (**B**) ciprofloxacin, (**C**) 2,2-dipyridyl, (**D**) NaCl, or (**E**) ethanol.

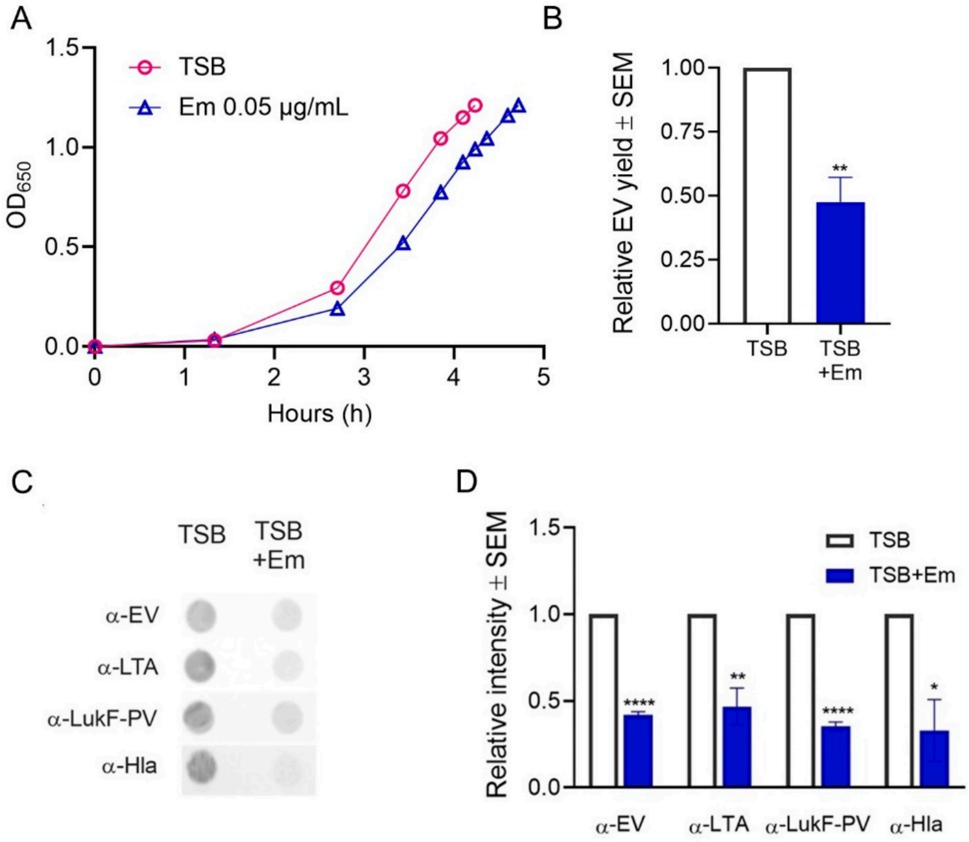

Figure S4. The effect of erythromycin on *S. aureus* EV production. (**A**) *S. aureus* was cultivated in 100 ml TSB with or without 0.05 μg/ml of erythromycin (Em) until an OD of 1.2 was achieved. (**B**) EV production was evaluated by quantification of relative EV protein yield or (**C**) by dot immunoblots of EV suspensions probed with indicated antibodies. Dot bots were performed at least three times, and a representative image is presented. (**D**) Relative intensity of dot blot images pooled from 3 independent experiments are shown. *$P < 0.05$, ** $P < 0.01$, **** $P < 0.0001$, as determined by the Student *t* test.
